# How Many People Need Palliative Care and How Many Miss Out?

**DOI:** 10.1093/geroni/igaa057.786

**Published:** 2020-12-16

**Authors:** Esther Davis, Victoria Westley-Wise, Stephen Moules, Malcolm Masso, Greg Barclay, Zivai Nangati, Joanne Davis, Kathy Eagar

**Affiliations:** 1 University of Wollongong, Wollongong, New South Wales, Australia; 2 Illawarra Shoalhaven Local Health District, Wollongong, New South Wales, Australia

## Abstract

With an ageing population and growing burden of chronic disease, the number of people requiring palliative or end-of-life care (P&EOLC) is set to rise. It is imperative to develop a comprehensive understanding of the quality and equity of P&EOLC provision to promote sustainable health systems. The aim of this presentation is to report an observational study of N=3,171 decedents across a Local Health Network to: 1) estimate the proportion who need, access and miss out on P&EOLC in the last 12 months of life, and 2) identify differences by clinical and sociodemographic characteristics. Analysis was performed on multiple integrated datasets containing routinely collected health and mortality data. Estimation methods based on underlying cause of death were applied to determine those decedents who could potentially benefit from P&EOLC. Results identified potential benefit to 75% of decedents, of which 62% received P&EOLC and 13% missed out. Decedents aged 85 years or more and from a residential aged care facility showed the lowest proportion of access. Decedents with diagnosis of liver or kidney failure and dementia received more P&EOLC than were expected to benefit. Multivariate logistic regression identified that diagnosis and no other clinical or sociodemographic factor was significantly associated with likelihood of accessing specialist palliative care, with cancer showing highest likelihood and heart failure lowest likelihood. This research highlights the value of population-based estimates to provide a ‘whole of system view’ of quality and equity of P&EOLC, with ready translation for service planners around resource allocation for need and likely benefit.

